# EV-Call 120: A new-generation emergency medical service system in China

**DOI:** 10.2478/jtim-2023-0143

**Published:** 2024-05-21

**Authors:** Puguang Xie, Liling Deng, Yu Ma, Wuquan Deng

**Affiliations:** Department of Endocrinology & School of Medicine, Chongqing University Central Hospital, Chongqing University, Chongqing 400014, China

## To the editor

**Figure 1:**
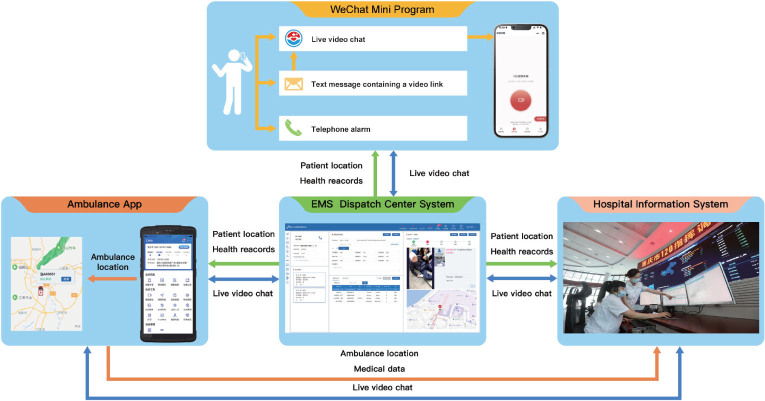
The flowchart of EV-Call 120.

Currently, healthcare systems worldwide are becoming increasingly reliant on remote medical services, especially the coronavirus disease 2019 (COVID-19) pandemic has led to a greater reliance on telehealth and accelerated the development of novel tools to improve the emergency medical efficiency and rescue ability.[^1, 2, 3, 4^] In recent years, research on the development and utility of real-time videos to support medical emergencies has emerged, and several video technology devices have been developed and applied to the clinical emergencies in many countries, such as Haven by RapidSOS in the United States and Alerter in UK.^[5, 6, 7, 8]^ Real-time videos and accurate locations could be achieved by remote services. However, the application of the above services is not specific to prehospital emergency medical system (EMS), remote live video medical guidance could not be achieved, and there is a scarcity of analyses on the effects of their applications. In response to these challenges, we have designed and developed a novel intelligent EMS system, named EV-Call 120 (Figure 1). This system diverges from existing medical dispatcher systems by offering multi-modal alarm and information transmission methods, encompassing voice, video, image and text. Furthermore, EV-Call 120 facilitates interaction with expert clinicians located within hospitals. Such a feature enables these clinical specialists to provide guidance and formulate comprehensive care plans for patients prior to their arrival at the hospital.

**Figure 2:**
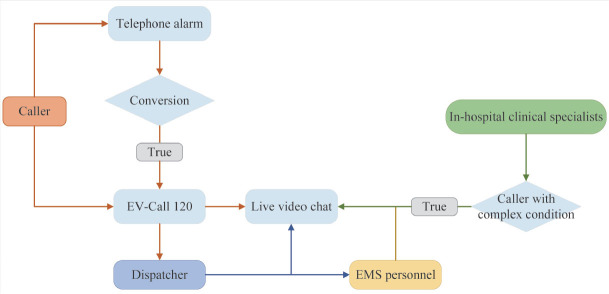
Schematic diagram of the application of EV-Call 120.

The EV-Call 120 system incorporates several advanced technological features including Web Real-Time Communication. This enables cross-platform, non-plugin video communication. The system also boasts effective network confrontation capabilities that ensure reliable communication even in environments with weak network signals. Moreover, it utilizes a targeted environmental sound noise reduction mechanism that helps maintain clear and quality communication in noisy surroundings. Utilizing hybrid positioning technology, the system allows for precise patient location tracking. Furthermore, multi-tag scheduling routing has been implemented in the system to facilitate efficient task allocation and resource management. These features collectively increase the system’s versatility and effectiveness. EV-Call 120 comprised four main components for the transmission of telemedicine information: a mobile WeChat mini program, an EMS dispatch center system, a hospital information system, and an ambulance application. In emergency situations, users have the ability to initiate a video call with the dispatcher via the WeChat mini program or mobile short messaging service link. In the course of this interaction, the program autonomously identifies the location of the caller, subsequently transmitting this data along with health information furnished by the user within the program to the EMS dispatch center system. Furthermore, the system provides a platform for the exchange of texts, images, and videos between the user and the dispatcher, enhancing the quality and specificity of communication. Following an exhaustive evaluation of variables such as the caller’s location, current traffic conditions, and the target hospital’s capacity for treatment, the dispatcher designates an ambulance and transfers the patient’s information to the EMS personnel. This team then joins the video call to provide immediate guidance to the patient. In instances where the patient’s condition is deemed complex and falls beyond the evaluative and treatment capabilities of the EMS personnel, clinical experts from the hospital are integrated into the video call to guide patient care. Additionally, the system disseminates the caller’s information to the closest volunteer equipped with professional first aid training, thereby expediting the provision of immediate assistance. An illustrative example of the application of EV-Call 120 is depicted in Figure 2.

**Figure 3:**
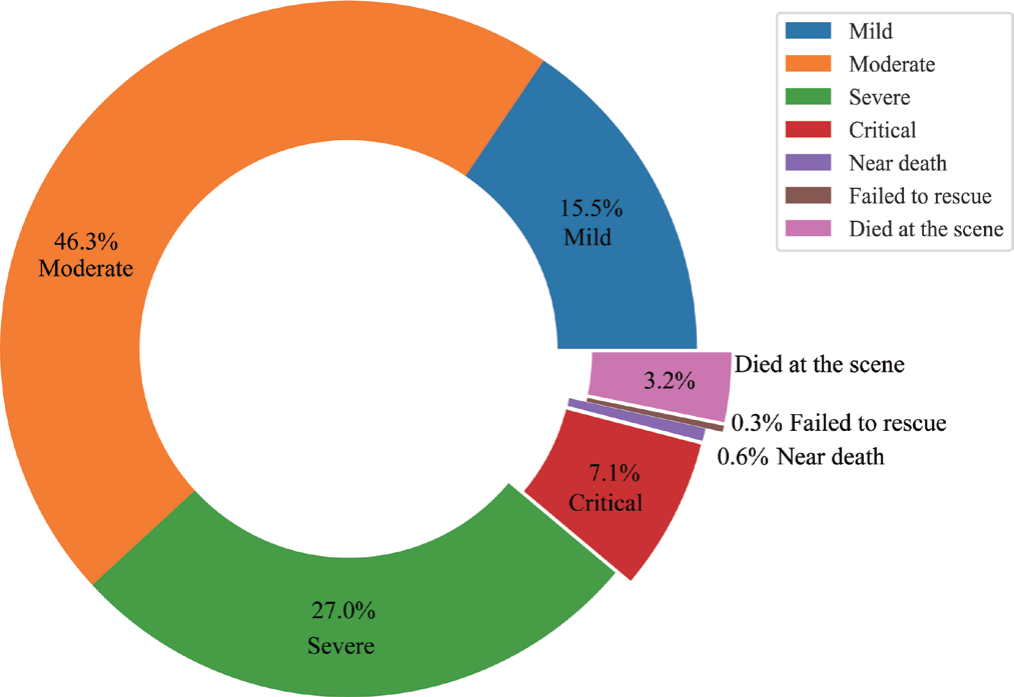
The proportion of patients with different severity levels in pre-hospital emergency care from August 1, 2021 to August 31, 2023.

The EV-Call 120 system was invented and introduced in Chongqing, a city in western China with a population of 32 million. This system was first launched and operated in August 2018 and we subsequently collected and analyzed operational data from August 1, 2021 to August 31, 2023. During this period, the city received a total of 3, 471, 054 EMS calls. A detailed analysis of patients with varying illness severity levels during this timeframe is presented in Figure 3. The EV-Call system was utilized 5, 339 times during this period. Thrillingly, post-video medical guidance, it was found that 64% of these patients no longer required ambulance services, greatly surpassing the 23% recorded for traditional telephone alarm. Thus, the EV-Call system significantly conserves invaluable medical resources. The traditional telephone alarm in the area had a first-aid response time of 13 to 16 min. However, with the introduction of the EV-Call 120, patients were now able to receive immediately, professional medical guidance, significantly improving response times. Furthermore, the EV-Call 120 system enhanced the timeliness and effectiveness of treatment delivery, especially for those in critical care. Patients in critical conditions requiring specialized hospital care were directly transferred to the recommended specialist hospital via the EV-Call 120 system. This facilitated timely and definitive treatment, consequently reducing the frequency of referrals.

To date, the EV-Call 120 system has been successfully deployed across various regions in China, establishing a novel paradigm for EMS. This has resulted in a significant reduction in first-aid response times. It has also driven the transition from singular audio communication towards multiple modes that incorporate real-time video, image, and text communication. Additionally, it has facilitated the shift of ambulance dispatch protocols from predominantly human decision-making to a more efficient, computer-assisted decision-making approach. Despite these advancements, a degree of instability in network signals has been observed in a few regions. This underscores the need for future improvements in platform reliability to minimize communication errors. The continued evolution, promotion, and application of the EV-Call 120 system are crucial to extend these benefits to a larger patient population. This ongoing development will not only optimize EMS but also enhance the medical emergency effectiveness of patients nationwide.
